# Low-density lipoprotein apheresis for recurrent focal segmental glomerulosclerosis in pediatric kidney transplant recipients: a systematic review and meta-analysis

**DOI:** 10.1007/s00467-025-07143-z

**Published:** 2026-02-11

**Authors:** Emily T. Hayes, Debora Matossian, Annie B. Wescott, Priya S. Verghese

**Affiliations:** 1 Department of Physiology and Biophysics, University of Illinois at Chicago, Chicago, IL USA; 2 Division of Nephrology, Ann & Robert H. Lurie Children’s Hospital of Chicago, Chicago, IL USA; 3 Department of Pediatrics, Northwestern University Feinberg School of Medicine, Chicago, IL USA; 4 Galter Health Sciences Library, Northwestern University Feinberg School of Medicine, Chicago, IL USA

**Keywords:** Recurrent FSGS, LDL-apheresis, Transplant

## Abstract

**Background:**

Recurrent focal segmental glomerulosclerosis (rFSGS) is a significant cause of graft failure in pediatric patients. Low-density lipoprotein apheresis (LDL-A) is an FDA-approved treatment for pediatric FSGS, but its efficacy is unclear.

**Objectives:**

This systematic review and descriptive meta-analysis aimed to determine the efficacy of LDL-A in pediatric kidney transplant recipients with rFSGS.

**Data sources:**

We performed a comprehensive search in Ovid MEDLINE, Cochrane Central Register of Controlled Trials (CENTRAL), Embase (Elsevier), CINAHL (EBSCO), and Scopus (Elsevier) on May 14th, 2024.

**Study eligibility criteria:**

Studies deemed eligible to be included were case reports, case series, randomized controlled trials, non-randomized controlled trials, and observational studies that reported patient-level data for subjects less than 18 years old who were administered any protocol of LDL-A following FSGS recurrence post-kidney transplant, and that provided remission status and urine protein–creatinine ratio (UPCR) ranges or values from at least one follow-up after LDL-A initiation.

**Participants and interventions:**

From the 8 studies that met the inclusion criteria, there were 25 patients who received LDL-A following rFSGS diagnosis post-transplant who were included for meta-analysis.

**Study appraisal and synthesis methods:**

Each study was assessed for selection bias, attrition bias, reporting bias, publication bias, and funding conflicts. The remission status for each patient was determined by the UPCR measured at the latest follow-up reported. Complete remission was defined as UPCR ≤ 0.2 g/g, partial remission as UPCR between 0.2 and 2.0 g/g, and no remission as UPCR ≥ 2.0 g/g. For our main outcome, the proportions of patients that achieved complete or partial remission were determined by study, then pooled estimates of effect size were calculated using a random-effects inverse-variance model. As a secondary outcome, the average effects of LDL-A on measures of kidney function were quantified by determining the median across individual changes in serum albumin, serum creatinine, estimated glomerular filtration rate (eGFR), and UPCR. Finally, subgroup analyses comparing remissions between LDL-A protocols were performed using Fisher’s exact test.

**Results:**

The pooled proportion of patients that achieved complete remission or partial remission was 0.36 (95% confidence interval (CI), 0.13–0.61) and 0.37 (95% CI, 0.14–0.62), respectively, at a median follow-up duration of 8 months (IQR 6–24 months) after LDL-A initiation. Median serum albumin and eGFR values were increased following LDL-A while UPCR decreased, consistent with clinical improvement. No significant differences in remissions were detected between LDL-A protocols, though the detection of real effects may be limited due to small sample sizes and heterogeneity.

**Limitations:**

All the included studies have moderate/high risk of bias due to study type, report type, and sample size. There is substantial variability between LDL-A protocols and previous treatments received by patients, possibly contributing to heterogeneity in outcomes between studies.

**Conclusions and implications of key findings:**

LDL-A achieved a complete remission rate of 36% (95% CI, 0.13–0.61) and a partial remission rate of 37% (95% CI, 0.14–0.62). Despite limited cases, LDL-A may be effective for pediatric rFSGS post-kidney transplant, warranting studies on its early use post-transplant.

**Systematic review registration number:**

PROSPERO (ID CRD42024544869).

**Graphical abstract:**

**Figure:**
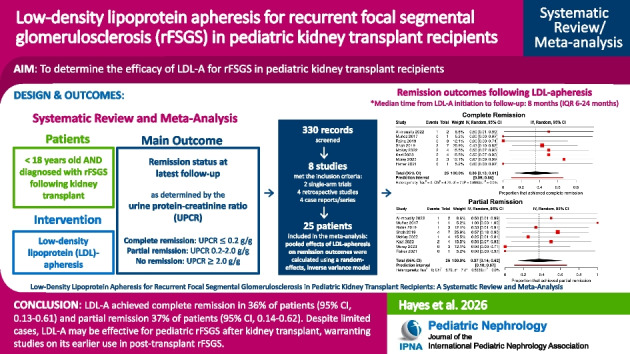
A higher resolution version of the Graphical abstract is available as Supplementary information

**Supplementary Information:**

The online version contains supplementary material available at 10.1007/s00467-025-07143-z.

## Introduction

Focal segmental glomerulosclerosis (FSGS) is estimated to be responsible for 11.5% of pediatric cases of kidney failure (also termed end stage kidney disease) in the United States [1]. Kidney transplant, the best strategy to treat almost all patients with kidney failure, is complicated in FSGS patients due to an unacceptably high risk of recurrent disease. In pediatric kidney transplant recipients that develop recurrent FSGS (rFSGS), 29–34% have graft failure as a result [1].

Since the identification of this disease as early as the 1970s, primary or “idiopathic” FSGS has been theorized to be mediated by an elusive circulating serum factor [2, 3]. Primary FSGS precludes any FSGS that is secondary to causes not limited to genetic mutations, metabolic disease, infection/inflammation-induced, hyper-filtration, or drug-induced causes [4].

For rFSGS that is primary, there is no proven prophylaxis, no standard treatment protocols, and no consensus guidelines, resulting in varying protocols and combinations of plasmapheresis, rituximab, and corticosteroids that vary by center [5–7]. Though generally efficacious in treating rFSGS, patients that fail to respond to first-line and second-line therapies have limited options, highlighting the need to comprehensively study promising alternative therapies. Through both designing clinical studies and synthesizing the data that is already available, headway can be made in determining the general efficacy of candidate alternatives in rFSGS, the characteristics of patients associated with positive response to treatment, and the efficacy of different treatment protocols and therapeutic combinations. Ultimately, studying alternative therapies stands to expand treatment options for rFSGS, especially for those with few options.

Recently, a promising but infrequently used alternative therapy called low-density lipoprotein apheresis (LDL-A) has been used to treat rFSGS in kidney transplant recipients. LDL-A is a non-surgical procedure that utilizes dextran sulfate cellulose adsorption to remove lipoproteins from the blood [8]. LDL-A is hypothesized to be therapeutic by reducing high levels of lipids in the blood that are thought to play a role in the pathogenesis of FSGS [9]. However, due to the lack of data and consensus on the efficacy of LDL-A for rFSGS, there are currently no standardized guidelines and protocols for practitioners to follow for using LDL-A to treat rFSGS in the pediatric kidney transplant population.

In the pediatric kidney transplant recipient population, there are few studies with small sample sizes reporting outcomes following varying protocols of LDL-A. Here, a systematic review and descriptive meta-analysis of this literature was performed to more comprehensively evaluate the efficacy of LDL-A in promoting remission in pediatric kidney transplant recipients with rFSGS.

## Methods

### Search strategy

The protocol for this systematic review was registered with PROSPERO (ID CRD42024544869). This study was performed in accordance with the PRISMA 2020 guidelines. A comprehensive search of the literature was conducted in partnership with a research librarian (A.W.). The search combined database-specific controlled vocabulary and title/abstract keywords related to LDL-apheresis in pediatric populations with FSGS. The search was designed and tested in Ovid MEDLINE and adapted to the following databases: Cochrane Central Register of Controlled Trials (CENTRAL), Embase (Elsevier), CINAHL (EBSCO), and Scopus (Elsevier). The full search strategy is available in Supplemental Table S5. All searches were conducted on May 14th, 2024, from database inception, without the use of filters or limits. Furthermore, handsearching via Google was performed to identify conference abstracts not indexed in traditional databases. All results were downloaded to a citation management software (EndNote) for multi-pass deduplication, and unique records were uploaded to an online screening platform (Rayyan) for independent screening.

### Eligibility criteria

Eligible studies that were included were published peer-reviewed articles and published abstracts of case reports, case series, randomized controlled trials, non-randomized controlled trials, and observational studies (cross-sectional, case–control, or cohort studies) that reported outcomes for pediatric patients (<18 years old) with recurrent FSGS following kidney transplant that were treated with LDL-A. The outcomes reported had to include remissions and degree of proteinuria. Non-English articles were translated to English by extracting the text from PDFs using optical character recognition via i2OCR (Sciweavers LLC, www.i2OCR.com) then translating the text using ChatGPT 5 (OpenAI, www.chatgpt.com) and Google Translate (Google, www.translate.google.com). Articles that met the inclusion criteria were independently reviewed for their eligibility by two investigators (E.H. and P.V.), and discrepancies were resolved by a third investigator (D.M.).

### Risk of bias assessment

The risk of bias for each included study was assessed using a customized framework based on established criteria for non-comparative studies, incorporating elements from the Joanna Briggs Institute critical appraisal tool [10] and guidance from the Cochrane Handbook for Systematic Reviews of Interventions [11]. Each study was assessed qualitatively for selection bias, attrition bias, reporting bias, publication bias, and funding conflicts, then the overall risk was assigned as low, moderate, or high. One reviewer assessed each study.

### Data extraction

A standardized data collection form was used to compile the following information from each included patient: study title, first author name, publication year, country, study type, patient sex, patient age at time of initial diagnosis and at time of transplant, transplant donor type, time from transplant to FSGS recurrence, treatments received for FSGS recurrence prior to LDL-A initiation, time from transplant to LDL-A initiation, LDL-A protocol description and characteristics including number of LDL-A sessions, the duration of LDL-A treatment, and any concomitant treatments, and any adverse events related to LDL-A treatment. For each patient, the following were extracted from each study for serum creatinine, serum albumin, eGFR, and urine protein–creatinine ratio (UPCR): final follow-up duration from the time of LDL-A initiation, numerical or categorical values for lab values at LDL-A initiation and at final follow-up, and any written descriptions of outcomes. If outcome data was presented only in plots, numerical values were extracted using PlotDigitizer (www.plotdigitizer.com). Data was only collected for patients <18 years old with recurrent FSGS after receiving a kidney transplant, while data from other patients in included studies were not extracted. One reviewer collected data from each report. No authors were contacted to request additional data.

### Synthesis methods

Due to the lack of comparative studies, a descriptive analysis was performed. Median and interquartile range (IQR) were used to report averages.

For certain values extracted for inclusion in the meta-analysis, there were differences between studies in whether certain data were reported as a number/continuous variable versus as a range/categorical variable; thus, conversion of variable types or exclusion of data was required for synthesis. To find the average time from transplant to LDL-A initiation, average number of sessions and duration of LDL-A, and the average duration from LDL-A initiation to final UPCR follow-up, values reported by studies as ranges were converted to a numerical value representing the median of the range (e.g., if the range “12–15” LDL-A sessions was reported by the study, “12–15” was converted to 13.5 for calculations), patients in which values were determined to be not reported by the original study were excluded for that specific calculation, then only the numerical values from each patient were compiled, and the median and interquartile range (IQR) were calculated.

For the main outcome of this study, we sought to determine the effect of LDL-A on rates of remission of proteinuria. To standardize remission status across studies, the numerical UPCR or categorical UPCR range reported as the final UPCR follow-up value for each patient was extracted and used to determine remission status based on the following definition: complete remission was defined as a UPCR of less than or equal to 0.2 g/g, partial remission was defined as a UPCR between 0.2 and 2.0 g/g, and no remission was defined as a UPCR of greater than or equal to 2.0 g/g, in line with the UPCR values specified in the definitions for complete remission, partial remission, and nephrotic-range proteinuria, respectively, in the KDIGO 2025 Clinical Practice Guideline for the Management of Nephrotic Syndrome in Children [12].

Forest plots were generated using MetaAnalysisOnline.com [13] to summarize the proportions of patients that achieved complete remission and partial remission by study and the pooled effect sizes. Each pooled effect size was calculated using a random-effects inverse-variance model and presented with a 95% confidence interval (95% CI). The 95% prediction intervals in the forest plots were calculated by MetaAnalysisOnline.com using the pooled estimate and between-study variance (Tau^2^) from the random-effects model. The certainty of evidence for remission rates—the primary outcome—was assessed using a qualitative approach based on risk of bias, consistency of results, indirectness of evidence, imprecision, and potential publication bias.

One of our secondary outcomes aimed to determine quantitatively the effect of LDL-A on four parameters of kidney function. For serum albumin levels, only patients with a numerical value reported for duration from LDL-A initiation to final follow-up for serum albumin, initial measurement of serum albumin at LDL-A initiation, and final measurement at final follow-up for serum albumin, were included for calculating the delta for serum albumin. Delta for each patient was determined by subtracting the initial from the final serum albumin measurement, then using that difference from each patient to calculate the median delta and IQR. The steps outlined in this paragraph were also used to calculate the change in serum creatinine, eGFR, and UPCR.

Further analyses were conducted to determine whether specific LDL-A protocol characteristics were associated with significant differences in remission rates. Remission outcomes were determined by separating patients into categories based on LDL-A protocol characteristics. Within each category, the number of patients who did not achieve remission (“no remission”) and the number of patients who achieved complete or partial remission (“any remission”) were determined and divided by the total number of patients in the category; this proportion was multiplied by 100 to get a percentage. To assess for significant differences in remissions between two categorical variables, a Fisher’s exact test was used due to small sample sizes and was performed using the number of patients per combination of remission status/protocol category. Significant differences between two medians were determined by the Mann–Whitney *U* Test. Statistical significance was defined as *p* < 0.05.

## Results

**Fig. 1:**
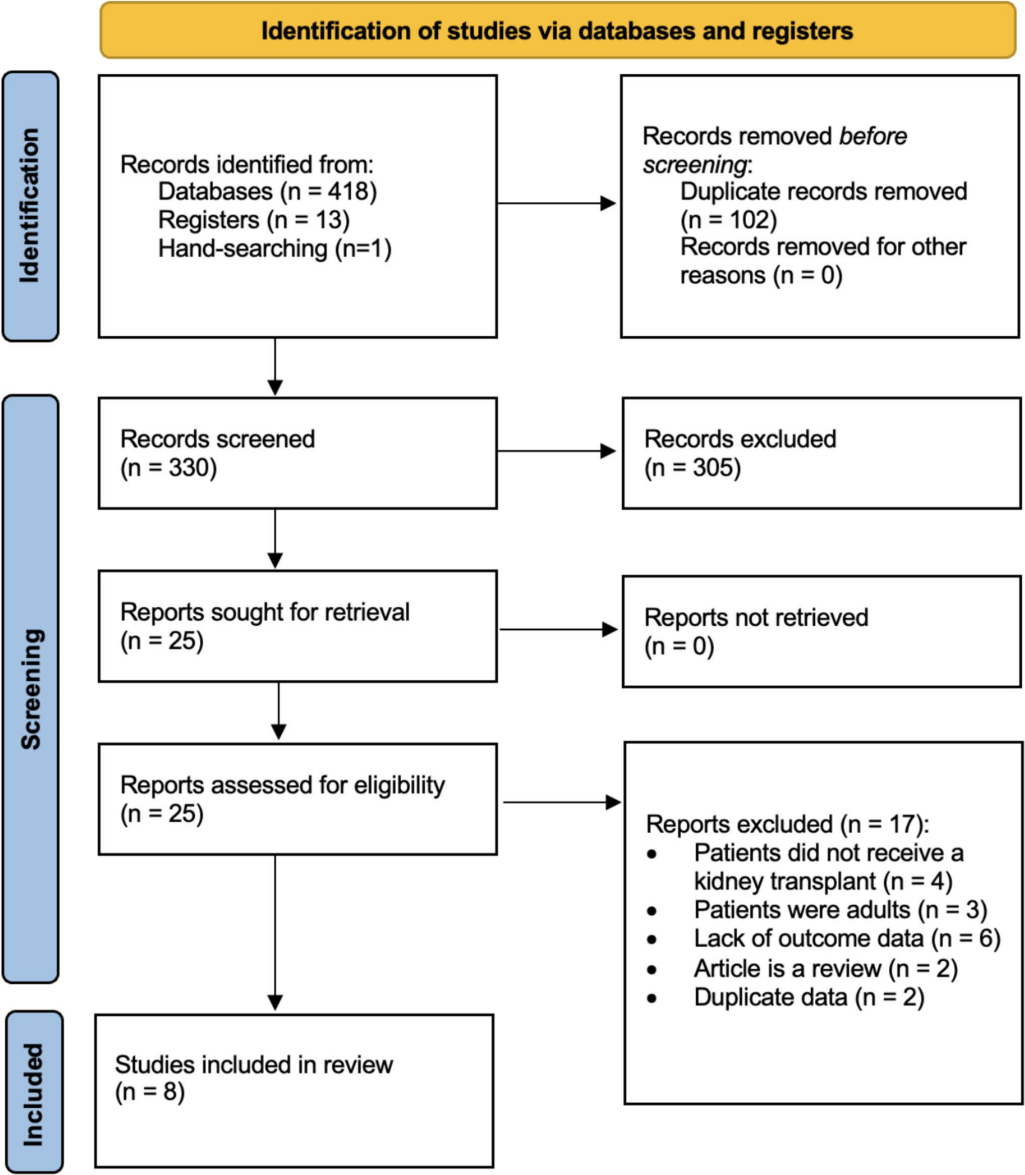
PRISMA 2020 flow diagram for study selection

Our search strategy in combination with hand-searching yielded 330 records after excluding duplicates (Fig. 1). Screening abstracts for the eligibility criteria resulted in the exclusion of 305 records, thus 25 reports were sought for retrieval. After a full-text review of the remaining records, 17 were excluded. Exclusions were made based on patient population, interventions, outcome data, article type, or reused data. Together, 8 studies were included in the review.

Of these 8 studies, 4 were conference abstracts and 4 were peer-reviewed articles (Table 1). The study types included 1 case study and 3 case series, 2 single-arm trials, and 2 retrospective single-center studies. All the included studies were published between 2017 and 2023. The studies were conducted in the United States, Canada, United Kingdom, Israel, and Spain. Data was extracted for each patient presented in the studies. However, only 3 patients in the study by Raina et al. met our inclusion criteria of having rFSGS post-transplant in addition to having follow-up data containing a UPCR [16]. One patient in the study by Kazi et al. was excluded from our meta-analysis due to presenting with de novo FSGS post-transplant [19]. Finally, 7 patients were excluded from the study by Fisher et al. due to not receiving LDL-A [21].

**Table 1:** Characteristics of included studies and summary of LDL-apheresis (LDL-A) protocol features by study

First author, year [reference], *N*	Country where study was performed	Study type, report type	Time from transplant to LDL-A initiation (range in months)	Sessions of LDL-A (range in number of sessions)	Duration of LDL-A treatment (range in weeks)	LDL-A protocol included pulse IV steroids (yes or no)	Duration from LDL-A initiation to UPCR final follow-up (range in months)
Al-mousily, 2022 [14] *N* = 2	United States	Case series, peer-reviewed article	0.25–2	12	9	No	6–36
Muñoz, 2017 [15] *N* = 1	Spain	Case study, conference abstract	36	12	9	Yes	24
Raina, 2019 [16] *N* = 3	United States	Single-arm trial, peer-reviewed article	Not reported	12	9	No	1–24
Shah, 2019 [17] *N* = 7	United States, United Kingdom	Single-arm trial, peer-reviewed article	0.25–18	12–28	9–22	Yes	4–25
McKay, 2022 [18] *N* = 4	Canada	Case series, conference abstract	0.1–20	12–15	9	Yes	11–19
Kazi, 2023 [19] *N* = 4	United States	Retrospective, single-center study, conference abstract	0–40	12–24	9–18	No	1–30
Morey, 2023 [20] *N* = 3	United States	Case series, conference abstract	Not reported	29–84	44–124	No	31–50
Fisher, 2021 [21] *N* = 1	Israel	Retrospective, single center study, peer-reviewed article	Not reported	10	20	Yes	15
Median (IQR, ***N***)	N/A	N/A	3 (0.25–14.75, ***N*** = 18)	13.5 (12–18.5, ***N*** = 25)	9 (9–16, ***N*** = 25)	N/A	8 (6–24, ***N*** = 25)

Each row summarizes key characteristics of the included publications, including study location, type, and details of LDL-A treatment protocols. Reported ranges represent the minimum and maximum values described per study. Medians and interquartile ranges (IQR) were calculated across all available data

Several of the reports assessed for eligibility appeared to meet the criteria but were excluded. Of the studies that described pediatric patients with rFSGS following kidney transplant that were treated with LDL-A, some were excluded due to the lack of required outcome data (remission status and UPCR after LDL-A initiation) [22–26] or data not being reported for individual patients [27]. There were other reports that met the inclusion criteria that contained the same data as one another, so the older reports were excluded [28, 29] and the newer reports were included [16, 20].

The overall risk of bias in the included studies was evaluated based on scoring the potential for selection bias, attrition bias, reporting bias, publication bias, and funding conflict; determined risk score (recorded as a heatmap, with low risk represented as a green circle, moderate risk represented as an orange circle, and high risk represented as a red circle) and summary of factors driving the risk score for each of the five bias domains are detailed by study in Supplemental Table S1. Of the 8 included studies, 3 were determined to have an overall high risk of bias, and 5 were determined to have a moderate risk of bias. This is in large part due to the nature of the types of studies (all studies are case series/reports, retrospective studies, and single-arm trials) and report types (4/8 being conference abstracts) that met the inclusion criteria (Table 1).

A total of 25 patients were included in this meta-analysis; individual data and summary statistics of patient characteristics are listed in Supplemental Table S2. The median age of patients at initial FSGS diagnosis was 3.5 years (IQR 2.0–6.3 years, *N* = 14), and the median age at the time of kidney transplant was 10 years (IQR 7–11.5 years, *N* = 19). The sex of the patients was 24% male, 36% female, and 40% not reported. The transplanted kidney received prior to FSGS recurrence came from a living donor for 3/25 patients (12%), deceased donor for 11/25 patients (44%), and the type of donor was not reported for 11/25 patients (44%). The time to FSGS recurrence following transplant was immediate (defined as less than 2 weeks after transplant) for 20 of the 25 patients (80%), not reported for 4 patients (16%), and delayed for 1 patient (4%) in which the time to recurrence for this patient was 40 months.

The LDL-A protocol characteristics by study with overall summary statistics are listed in Table 1 and detailed by patient in Supplemental Table S3. The specific equipment used for LDL-A, reported by 4 of the included studies, was the Liposorber LA-15 System, produced by Kaneka Medical America LLC. The time from transplant to LDL-A initiation was a median of 3.0 months (IQR 0.25–14.75 months, *N* = 18; Supplemental Table S3). Though the time from transplant to treatment differs from measuring the time from the onset of FSGS recurrence, 80% of patients in this analysis experienced immediate recurrence (Supplemental Table S2), thus using the time from transplant to LDL-A—which was more consistently reported/determined across studies—likely only overestimates the time from disease onset to LDL-A by up to 2 weeks for the majority of the patients in this analysis. With the median time to LDL-A treatment being 3.0 months (Supplemental Table S3), this delay likely represents time in which first-line and second-line therapies for rFSGS were attempted, consistent with 23/25 patients (92%) having received some combination of treatments—including at least 1 first-line rFSGS therapy—for recurrence prior to initiating LDL-A (Supplemental Table S2).

The studies by Al-mousily et al., Raina et al., Kazi et al., and Morey et al. utilized an LDL-A protocol in which patients received 2–3 sessions of LDL-A for the first 3 weeks, followed by 1 session per week for the remaining 6 weeks, for a total of 12–15 sessions of LDL-A over 9 weeks (Table 1). However, 1 patient in the study by Kazi et al. had a clinical relapse 4 months after the first round of LDL-A and underwent a second round of LDL-A for a total of 24 sessions (Supplemental Table S3) [19]. Further, all 3 patients in the study by Morey et al. continued with approximately weekly LDL-A sessions after the completion of the first 12, receiving a total of 29–84 LDL-A sessions over 44–124 weeks (Table 1, Supplemental Table S3). The studies by Muñoz et al., Shah et al., and McKay et al. sought to deploy a protocol in which patients receive 2–3 LDL-A treatments each week for the first 3 weeks, followed by 1 LDL-A session per week in addition to pulse IV steroids, specifically IV methylprednisolone at a dose of 10–20 mg/kg for a single maximum dose of 1 g, totaling 12–15 LDL-A treatments over 9 weeks. However, 3 of the 7 patients in the study by Shah et al. were continued on LDL-A treatment after the initial 12–15, receiving a total of 17–28 LDL-A sessions over 14–22 weeks (Table 1, Supplemental Table S3). Finally, the patient from the study by Fisher et al. received an LDL-A session once every 2 weeks in combination with high-dose IV steroids for a total of 10 sessions over 20 weeks, though the protocol in this study was not described further. Across all included studies and patients, there was a median total of 13.5 LDL-A sessions (IQR 12–18.5 sessions) administered over a median duration of 9 weeks (IQR 9–16 weeks; Table 1). For the main outcome remission status at final/latest follow-up when the latest reported UPCR value was measured–the median final follow-up duration was 8 months (IQR 6–24 months; Table 1).

Adverse events related to LDL-A treatment were only discussed or mentioned in 3 of the included studies. In the study by McKay et al., the complications reported were BK viremia, neutropenia, retroperitoneal hemorrhage, common femoral vein thrombus, and Candida bacteremia [18]. Kazi et al. reported no adverse effects [19]. Finally, the study by Raina et al. reported the following side effects: nausea, vomiting, diarrhea, abdominal pain, fever/infection, pharyngitis, headache, lightheadedness, malaise, hypotension, leg cramps, allergic reaction, pneumonia, bacteremia, and anemia [16].

**Fig. 2:**
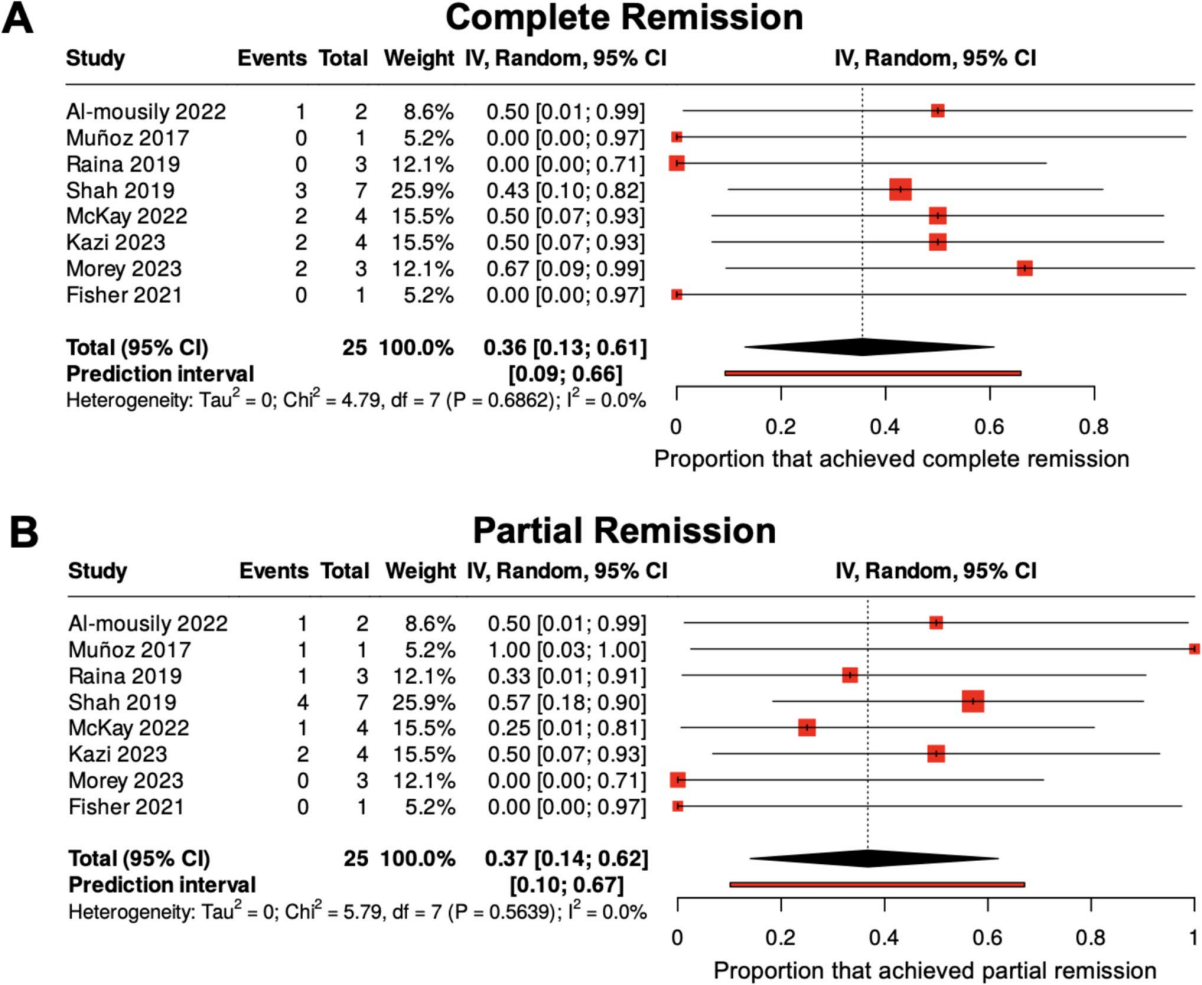
Forest plots of remission outcomes following LDL-apheresis for recurrent FSGS. Each forest plot shows the proportion of patients that achieved complete remission (**A**) or partial remission (**B**) in each included study, with 95% confidence interval (CI) and study weight (“Weight”) derived from a random-effects inverse-variance (“IV, Random”) model. The diamond represents the pooled estimate of effect size with its 95% CI. Prediction intervals indicate the expected range of treatment effects in future comparable studies. Heterogeneity statistics (Tau^2^, Chi^2^, *P* value, I^2^) are reported beneath each plot

The primary outcome of this study was to determine the effect of LDL-A on rates of remission of proteinuria in pediatric patients with FSGS recurrence post-transplant. Remission status was determined for all 25 patients by extracting the numerical UPCR values, or extracting categorical UPCR ranges based on individual study definitions, for the latest follow-up reported for each patient. Remission status, recorded by patient in Supplemental Table S4, was determined based on the following definitions: “complete remission” was defined as UPCR ≤ 0.2 g/g, “partial remission” as UPCR between 0.2 and 2.0 g/g, and “no remission” as UPCR ≥ 2.0 g/g. The number and proportion of patients by study that achieved complete remission or partial remission are summarized in the forest plots in Fig. 2. To estimate the effect size of LDL-A on remission rates while accounting for between-study heterogeneity and incorporating study weighting, a random-effects inverse-variance model was used to calculate pooled proportions. The pooled proportion of patients achieving complete remission (Fig. 2A) or partial remission (Fig. 2B) was 0.36 (95% CI: 0.13–0.61) and 0.37 (95% CI: 0.14–0.62), respectively. This indicates that, on average, 36% of patients achieved complete remission and 37% achieved partial remission following LDL-A treatment, with the 95% prediction intervals presented in Fig. 2 suggesting similar future studies would plausibly see rates of complete remission between 9 and 66% and rates of partial remission between 10 and 67% following LDL-A treatment. The I^2^ and Tau^2^ values—two different statistics to estimate heterogeneity between studies—were 0.0% and 0, respectively, as calculated for both the rates of complete remission (Fig. 2A) and partial remission (Fig. 2B), both suggesting that there is no observed variability between studies and consistency between study effect sizes. Thus, the estimated effect sizes are unlikely to be due to heterogeneity and more likely to be a real effect of the intervention. However, this interpretation is limited by the small number of included studies and small sample sizes.

As a secondary outcome, we aimed to quantify the effects of LDL-A on kidney function by calculating changes in serum albumin, serum creatinine, eGFR, and UPCR. Lab values at LDL-A initiation (baseline/initial values) and the latest values reported/measured at the final follow-up for a given parameter were extracted for each of the 25 patients; lab values are listed by patient in Supplemental Table S4. If the patient had both final and initial values reported, the difference between final and initial measurements (“delta”) was calculated for each patient, then the median difference across patients was determined, as summarized in Table 2. For serum albumin, there was a median increase of 1.1 g/dL (IQR 0.7 to 1.8 g/dL) from a median 2.6 g/dL (IQR 2.4 to 2.8 g/dL) at baseline to a median final lab value of 3.9 g/dL (IQR 3.5 to 4.3 g/dL) over a median final follow-up duration of 7.2 months (IQR 6.0 to 28.5 months, *n* = 14). This substantial increase brought the average of hypoalbuminemia levels back to the normal range at long-term follow-up durations, suggesting a meaningful improvement in the amount of protein being lost in the urine and maintained in circulation. Serum creatinine showed no change (median 0.0 mg/dL, IQR –1.1 to 0.3 mg/dL, *n* = 7), with both the initial and final median values being in the normal range, indicative of sustained graft function. The median initial eGFR was 47.7 mL/min/1.73 m^2^ (IQR 27.7 to 78.0 mL/min/1.73 m^2^, *n* = 17), a level indicative of moderate kidney dysfunction in line with FSGS recurrence. However, at a median follow-up duration of 16.0 months (IQR 4.0 to 25.0 months), the average increase in eGFR was 41.6 mL/min/1.73 m^2^ (IQR 5.0 to 54.2 mL/min/1.73 m^2^) with a median final eGFR of 81.1 mL/min/1.73 m^2^ (IQR 60.0 to 105.7 mL/min/1.73 m^2^). Thus, on average, there was a substantial recovery of graft function, consistent with partial remission of rFSGS. Finally, UPCR values demonstrated a median change of −6.8 g/g (IQR −17.8 to −3.0 g/g, *n* = 15) over a median final follow-up duration of 6.0 months (IQR 5.1 to 16.0 months, *n* = 15). This is a dramatic decrease from a median initial UPCR of 10.0 g/g (IQR 5.8 to 18.9 g/g)—severely in the nephrotic range—to an average final UPCR of 0.4 g/g (IQR 0.3 to 1.7 g/g), which falls towards the lower end of the UPCR range that defines partial remission (0.2–2.0 g/g). Together, this descriptive analysis demonstrated large, clinically meaningful changes in 3 parameters of kidney function (serum albumin, eGFR, and UPCR) at long-term endpoints (greater than 6 months on average) after LDL-A treatment.

**Table 2:** Change in serum albumin, serum creatinine, estimated glomerular filtration rate (eGFR), and urine protein-creatinine ratio (UPCR) between LDL-apheresis (LDL-A) initiation and final follow-up

	Serum albumin (***N*** = 14)	Serum creatinine (***N*** = 7)	eGFR (***N*** = 17)	UPCR (***N*** = 15)
Duration from LDL-A initiation to final follow-up	7.2 months	2.3 months	16.0 months	6.0 months
Median (IQR)	(6.0–28.5 months)	(1.5–24.0 months)	(4.0–25.0 months)	(5.1–16.0 months)
Initial lab value	2.6 g/dL	0.8 mg/dL	47.7 mL/min/1.73 m^2^	10.0 g/g
Median (IQR)	(2.4–2.8 g/dL)	(0.7–1.6 mg/dL)	(27.7–78.0 mL/min/1.73 m^2^)	(5.8–18.9 g/g)
Final lab value	3.9 g/dL	0.6 mg/dL	81.8 mL/min/1.73 m^2^	0.4 g/g
Median (IQR)	(3.5–4.3 g/dL)	(0.5–1.1 mg/dL)	(60.0–105.7 mL/min/1.73 m^2^)	(0.3–1.7 g/g)
Delta (Final lab value-initial lab value)	1.1 g/dL	0.0 mg/dL	41.6 mL/min/1.73 m^2^	–6.8 g/g
Median (IQR)	(0.7–1.8 g/dL)	(–1.1 to 0.3 mg/dL)	(5.0–54.2 mL/min/1.73 m^2^)	(–17.8 to –3.0 g/g)

Values are presented as median (interquartile range, IQR). “Delta” represents the change between final and initial laboratory values

Given the variations in LDL-A protocols across the included studies (summarized by study in Table 1 and by patient in Supplemental Table S3), we hoped this would allow for subgroup analyses to evaluate differences in efficacy between specific protocol features as a final secondary outcome. Specifically, we evaluated two main sources of variation between protocols: the incorporation of pulse IV steroids as a concomitant treatment (4/8 studies included concomitant pulse IV steroids in the protocol, 4/8 studies did not use pulse IV steroids), and the total number of LDL-A sessions administered per patient, ranging from a minimum of 10 to a maximum of 84 total LDL-A sessions (Table 1).

To determine whether the concomitant use of pulse IV steroids with LDL-A has an effect on remission rates, patients were separated into two categories based on whether the LDL-A protocol included pulse IV steroids (LDL-A with pulse IV steroids: *n* = 13, LDL-A, no pulse IV steroids: *n* = 12); then significant differences in the proportions of patients that achieved any remission—which includes both complete and partial remissions—or no remission were compared between categories by Fisher’s exact test. To test for possible confounding effects of other main LDL-A protocol variables on remissions, category medians for total LDL-A sessions, LDL-A duration, and duration to final UPCR follow-up (Table 3) were tested for significant differences between categories by Mann–Whitney *U* Test; no significant differences were detected. In the group administered protocols utilizing pulse IV steroids, 75% achieved any remission and 25% did not achieve remission. In the group that did not receive pulse IV steroids in the LDL-A protocol, 85% achieved any remission and 15% did not achieve remission. There was no significant difference in remission outcomes between categories.

**Table 3:** Remission outcomes by LDL-apheresis (LDL-A) protocol characteristics

LDL-A protocol feature being evaluated	LDL-A protocol feature sub-categories (N)	Sessions of LDL-A Median (IQR)	Duration of LDL-A Median (IQR)	Duration from LDL-A initiation to UPCR final follow-up Median (IQR)	Any remission (complete or partial remission),* n*/total (%)	No remission, *n*/total (%)
Use of concomitant pulse IV steroids	LDL-A, no pulse IV steroids (*N* = 12)	12.0 sessions (12.0–25.25 sessions)	9.0 weeks (9.0–24.5 weeks)	24.0 months (2.2–32.3 months)	9/12 (75)	3/12 (25)
	LDL-A, with pulse IV steroids (*N* = 13)	13.5 sessions (13.5–13.5 sessions)	9.0 weeks (9.0–14.0 weeks)	8.0 months (6.0–15.0 months)	11/13 (85)	2/13 (15)
The total number of LDL-A sessions	12–18.5 total LDL-A sessions (*N* = 18)	12.8 sessions (12.0–13.5 sessions)^a^	9.0 weeks (9.0–9.0 weeks)^b^	7.0 months (4.7–18.3 months)^c^	15/18 (83)	4/18 (17)
	>18 LDL-A sessions (*N* = 6)	27.8 sessions (24.6–38.0 sessions)^a^	33.0 weeks (19.0–50.0 weeks)^b^	27.5 months (11.6–36.3 months)^c^	5/6 (83)	1/6 (17)

Median number of LDL-apheresis (LDL-A) sessions, treatment duration, and follow-up are shown with interquartile ranges (IQR). “Any remission” includes complete or partial remission, defined. By a urine protein–creatine ratio (UPCR) ≤ 2.0 g/g at last follow-up. Categorical proportions (remission vs. no remission) were compared using Fisher’s exact test; no statistically significant differences were observed in rates of remission between categorical groups. Differences in median values were assessed using the Mann–Whitney *U* test; significant differences (*P* < 0.05) between two medians are indicated by shared superscript letters

Similarly, to assess whether the total number of LDL-A sessions affects remission rates, patients were separated into two categories based on whether the number of LDL-A sessions fell within the overall interquartile range for the total number of LDL-A sessions (Table 1) across all included patients (12–18.5 sessions, *n* = 18)—representing an average number of LDL-A sessions—or whether the number of sessions fell above the interquartile range (>18.5 sessions, *n* = 6), representing receiving an above-average number of LDL-A sessions. One patient was excluded due to the number of sessions falling below the interquartile range. As expected, there was a significant difference in the median number of LDL-A sessions between categories (12.8 vs. 27.8 sessions, *P* = 0.0004), in the median LDL-A duration between categories (9.0 weeks vs. 33.0 weeks, *P* = 0.0004), and in the median time to final follow-up (7.0 months vs. 27.5 months, *P* = 0.049). Rates of any remission (83%) and no remission (17%) were identical between categories (Table 3). These subgroup analyses detected multiple significant differences in parameter averages outside of the main comparison parameter, and also failed to detect differences in remission rates between categories of LDL-A protocol characteristics, suggesting that detection of real effects of specific LDL-A protocol features may be limited by small sample sizes and heterogeneity in patients, protocols, and outcomes.

For primary outcomes—rates of complete remission and partial remission—the overall certainty of evidence was determined by considering the risk of bias, consistency of results, indirectness of evidence, imprecision, and potential publication bias. The risk of bias, as discussed above, was moderate or high for all included studies due in large part to including study and report types that have a high potential for selection bias, reporting bias, and publication bias. Inconsistency was assessed using the I^2^ and Tau^2^ statistics calculated for the proportion of patients that achieved complete remission (I^2^ = 0.0%, Tau^2^ = 0; Fig. 2A) and partial remission (I^2^ = 0.0%, Tau^2^ = 0; Fig. 2B) across studies. These statistics suggest consistency and little heterogeneity between studies for rates of remissions, though these calculations are limited by the small number of studies and sample sizes. In considering the indirectness of evidence, the included studies and patients differed moderately in protocols and concomitant treatments, remission definitions, treatments for rFSGS prior to LDL-A initiation, and how remission outcomes were reported, and differed majorly in durations of follow-up and thus when remission status was determined. Imprecision was judged based on the size of the confidence intervals and sample sizes; confidence intervals for the effect of LDL-A on the proportion of patients that achieved complete remission (95% CI, 0.13–0.61; Fig. 2A) and partial remission (95% CI, 0.14–0.62; Fig. 2B) were wide and based on a small number of patients. Finally, the potential for publication bias was determined to be moderate due to half of the studies being non-peer-reviewed conference abstracts and half being case reports with positive outcomes. Together, our evidence was considered to have a high risk of bias, low inconsistency, moderate indirectness, high imprecision, and moderate potential for publication bias. Thus, our overall certainty of evidence was determined to be low, despite good statistical heterogeneity.

## Discussion

In this study, a systematic review and meta-analysis was performed evaluating the efficacy of LDL-A on treating rFSGS following kidney transplant in pediatric patients. Due to the rarity of data in this population using this intervention, this systematic review allows, for the first time, to evaluate the efficacy of LDL-A in a larger number of patients across several centers and countries. Our primary outcomes were the rates of remission of proteinuria following LDL-A. Using the 25 total patients compiled from the 8 studies that met the inclusion criteria, there was an estimated pooled proportion of patients that achieved complete remission of 0.36 (95% CI 0.13–0.61; Fig. 2A), and the estimated pooled proportion of patients that achieved partial remission was 0.37 (95% CI 0.14–0.62; Fig. 2B), at a median follow-up duration from LDL-A initiation of 8 months (IQR 6–24 months; Table 1). The 95% prediction intervals calculated in this analysis estimate that in similar studies, LDL-A would likely induce complete remission in 9–66% of patients (Fig. 2A), and partial remission in 10–67% of patients (Fig. 2B). Together, these effect sizes support the therapeutic promise for LDL-A in this population.

Further quantitative analyses were performed to determine the effects of LDL-A on several parameters of kidney function: serum albumin, serum creatinine, eGFR, and UPCR (Table 2). Strikingly, median final and initial serum albumin values improved from hypoalbuminemia levels at baseline to normal levels at final follow-up. Median eGFR at baseline fell into the chronic kidney disease (CKD) Stage 3a range and improved to a median eGFR that fell into CKD Stage 2. Finally, median UPCR at baseline, representing severe nephrotic syndrome, decreased 96% to the median UPCR at final follow-up, to levels representing partial remission of proteinuria. Together, these substantial changes in serum albumin, eGFR, and UPCR represent large, meaningful improvements in graft function on average following LDL-A treatment, further strengthening the therapeutic potential of LDL-A.

Finally, subgroup analyses were performed comparing remission outcomes to determine whether certain LDL-A protocol characteristics influenced rates of remission (Table 3). No significant differences in remissions were detected between protocol characteristics studied: the incorporation of pulse IV steroids as a concomitant treatment and the total number of LDL-A sessions administered. It is possible that the small sample sizes and heterogeneity of extracted data in this study may prevent the detection of real differences between LDL-A techniques.

There were several limitations in this descriptive meta-analysis. Namely, the small number of included studies and patients, the report types all having moderate to high risk of bias, the substantial variation in LDL-A protocols and reporting of outcomes, and the moderate confidence intervals all resulted in a low certainty of evidence for our calculated pooled effect sizes.

Further, there was a lack of comparison groups in the included studies to compare the efficacy of LDL-A to other first-line therapies, including rituximab, plasmapheresis, and immunosuppression. Because these first-line therapies are efficacious, it is unlikely that there will be a randomized controlled trial evaluating the efficacy of LDL-A alone versus other first-line therapies. In the same vein, most of the patients included in this meta-analysis received first-line treatments for rFSGS prior to initiating LDL-A; 23/25 patients received some combination of treatment that included at least one first-line treatment, 21/25 received plasmapheresis, 20/25 received rituximab or other B cell depletion therapy, and 23/25 received at least one immunosuppressive treatment (Supplemental Table S2). Given that the majority of patients in this analysis had nephrotic-range proteinuria at the time of LDL-A initiation, this suggests that this compiled cohort of patients may represent an even smaller subpopulation of pediatric patients with rFSGS post-kidney transplant that are resistant to the traditional first-line therapies, ultimately leading these patients to be trialed on LDL-A as an alternative approach after the traditional approaches failed to induce remission. With the difficulties and impracticalities apparent that would make the production of sufficiently powered high-quality evidence for LDL-A in this population unlikely and untimely, and with the lack of treatments available for patients resistant to first-line therapies, it is imperative to synthesize the data currently available despite its limitations and make appropriate conclusions to potentially establish LDL-A protocol guidelines and recommendations to treat patients resistant to other therapies.

Despite these limitations, our overall estimated pooled response rates of 36% and 37% of patients achieving complete and partial remission, respectively, are similar to the response rates reported by other studies that evaluated LDL-A efficacy in the context of refractory nephrotic syndrome in non-transplant adults and children. In a retrospective survey of 36 hospitals in Japan, 87% of the 41 adult patients with nephrotic syndrome treated with LDL-A were classified as complete or partial remission after 5 years [30]. In a systematic review and meta-analysis of the efficacy of LDL-A in adult patients with native kidney FSGS, 54% of the 61 total patients responded [31]. Finally, a case series of 5 pediatric patients with resistant nephrotic syndrome showed that 80% achieved complete or partial remission with LDL-A [32].

Though LDL-A will likely not be considered a first-line treatment in isolation for rFSGS due to the efficacy of established therapies, it is still important to compare the estimated pooled rates of remission found in our meta-analysis (complete remission: 36%, partial remission: 37%; Fig. 2) to the current first-line therapies: plasmapheresis and rituximab. A 2010 meta-analysis found that 49/70 (70%) of pediatric patients with rFSGS who received plasmapheresis or immunoadsorption, and 11/19 (58%) of patients (both adult and pediatric) who received rituximab achieved complete or partial remission [33]. Another meta-analysis evaluating the efficacy of plasmapheresis or rituximab in pediatric and adult rFSGS patients reported 36/59 patients (61%) achieved complete or partial remission with plasmapheresis, and 79% of rFSGS patients responded to rituximab [34]. Considering the estimated pooled rates of complete and partial remission together (36% and 37%, respectively), this suggests that LDL-A may have a similar response rate to first-line therapies like rituximab and plasmapheresis, further supporting the therapeutic potential of LDL-A for pediatric patients with rFSGS post-transplant that are resistant to other therapies. This also reveals exciting and important future directions to study the efficacy of first-line therapies in combination with LDL-A on rFSGS.

Adverse events related to LDL-A were only discussed by 3 of the 8 studies (in which 1 of those 3 studies reported no adverse events) and were generally mild, including transient mild hypotension, gastrointestinal symptoms, and nonspecific complaints such as malaise and fatigue [16, 18, 19]. Occasional thrombotic or infectious complications were also reported [18]. Compared to the typical first-line therapies for rFSGS, rituximab and plasmapheresis, which carry higher risks of significant adverse events including severe infusion reactions, cytopenias, and increased susceptibility to infections, LDL-A appears to be better tolerated.

Though LDL-A treatment showed efficacious rates of remission and average changes in kidney parameters indicative of improved graft function (Table 2), the variation between the LDL-A protocols across studies and patients was a significant limitation in being able to perform further analyses and detect true effects. Moving forward, standardization of LDL-A protocols and guidelines will be necessary to establish LDL-A as an effective, recommended treatment option to consider for rFSGS after or in addition to first-line therapies.

In conclusion, our data suggest that LDL-A demonstrates long-term efficacy in achieving partial or complete remission in pediatric patients with rFSGS following kidney transplant. Further, LDL-A may be effective in some recipients with rFSGS, especially for patients that do not respond to first-line treatments. Moving forward, LDL-A should be considered for inclusion in a multi-site case series with historical controls to further evaluate effectiveness. Further, LDL-A should be studied in combination with current first-line therapies to determine benefits or synergy in adding LDL-A. Finally, it is imperative that guidelines and LDL-A protocols be established to standardize the use of LDL-A in treating rFSGS in post-transplant pediatric patients across the world.

## Supplementary Information

Below is the link to the electronic supplementary material.

ESM1 (DOCX 16.9 KB)

ESM2 (DOCX 26.5 KB)

ESM3 (DOCX 18.4 KB)

ESM4 (DOCX 23.0 KB)

ESM5 (DOCX 15.0 KB)

ESM6 (PPTX 386 KB)
