# Supplementary material for: Low-density lipoprotein apheresis for recurrent focal segmental glomerulosclerosis in pediatric kidney transplant recipients: a systematic review and meta-analysis
Source: Pediatr Nephrol. 2026 Feb 11;41(9):2849–61. doi: 10.1007/s00467-025-07143-z (PMC13424331; doi:10.1007/s00467-025-07143-z)
Supplement: Supplementary file 1 — (DOCX 16.9 KB) [file 467_2025_7143_MOESM1_ESM.docx]

**Supplemental Table S1. Risk of bias assessment.**

| **Study first author last name, year** | **Selection Bias** | **Attrition Bias** | **Reporting Bias** | **Publication Bias** | **Funding Conflicts** | **Overall Risk of Bias** |
| --- | --- | --- | --- | --- | --- | --- |
| **Al-mousily, 2022** | 🔴 Unclear if all cases were reported or if selection was biased toward unique presentations. | N/A (case series). | 🔴 Only favorable outcomes reported. No mention of complications. | 🟠 Peer-reviewed, but only positive findings published. | 🔴 Senior author received funding from LDL-A equipment manufacturer. | **🔴 High** |
| **Muñoz, 2017** | 🔴 Case selected due to novelty, unclear if others were excluded. | N/A (case study). | 🔴 Limited details; only favorable outcomes reported. Some complications addressed. | 🔴 Published as a conference abstract only. | 🟠 No disclosure statement. | **🔴 High** |
| **Raina, 2019** | 🟠 Clear inclusion criteria, but recruitment process unclear. | 🔴 High dropout rate (27%) with unexplained attrition. | 🟢 All pre-specified outcomes reported. Complications addressed. | 🟢 Published in a peer-reviewed journal. | 🟢 No major funding conflicts disclosed. | **🟠 Moderate** |
| **Shah, 2019** | 🟠 Incomplete inclusion criteria. No exclusion criteria. Recruitment process unclear. | 🟠 Moderate attrition, but addressed in study. | 🟠 Limitations addressed, but no mention of complications. | 🟢 Published in a peer-reviewed journal. | 🟢 No major funding conflicts disclosed. | **🟠 Moderate** |
| **McKay, 2022** | 🟠 Consecutive cases reported, but unclear if all were included. | N/A (case series). | 🟢 Addressed complications and limitations. | 🔴 Published as a conference abstract only. | 🟠 No disclosure statement. | **🟠 Moderate** |
| **Kazi, 2023** | 🟠 Clear inclusion criteria, but unclear if selection was biased. | N/A (retrospective). | 🟢 All pre-specified outcomes reported. No mention of limitations. | 🔴 Published as a conference abstract only. | 🟠 No disclosure statement. | **🟠 Moderate** |
| **Morey, 2023** | 🔴 Unclear selection process. May not include all relevant cases. | N/A (case series). | 🔴 Complications incompletely addressed, no limitations discussed. | 🔴 Published as a conference abstract only. | 🟠 No disclosure statement. | **🔴 High** |
| **Fisher, 2021** | 🟠 Consecutive patients identified retrospectively from a single center, but unclear if all eligible cases were included. | N/A (retrospective). | 🟠 Reported both positive and negative outcomes, but limited discussion of individual complications and missing data. | 🟢 Published in a peer-reviewed journal | 🟢 No funding or conflict of interest disclosed. | 🟠 **Moderate** |

For each study that met the systematic review inclusion criteria (identified above by the first author’s last name and the year of the report’s publication in the first column), the overall risk of bias was determined by assessing the presence and severity of each of the following types/sources of bias: selection bias, attrition bias, reporting bias, publication bias, and funding conflicts. For each bias domain, a study was determined to have low, moderate, or high risk, indicated in the table above by a green, orange, or red circle, respectively, along with a summary of the driving factors for each determination.
