# Supplementary material for: Low-density lipoprotein apheresis for recurrent focal segmental glomerulosclerosis in pediatric kidney transplant recipients: a systematic review and meta-analysis
Source: Pediatr Nephrol. 2026 Feb 11;41(9):2849–61. doi: 10.1007/s00467-025-07143-z (PMC13424331; doi:10.1007/s00467-025-07143-z)
Supplement: Supplementary file 2 — (DOCX 26.5 KB) [file 467_2025_7143_MOESM2_ESM.docx]

**Supplemental Table S2. Individual and summary data of patient characteristics and prior treatments for all patients included in the meta-analysis.**

| **Patient** | **Study first author last name, year** | **Patient sex** | **Patient age at time of initial FSGS diagnosis (years)** | **Patient age at time of transplant (years)** | **Transplant donor type (living or deceased)** | **Time from transplant to FSGS recurrence (immediate or delayed)** | **Treatments received for FSGS recurrence prior to LDL-A initiation** |
| --- | --- | --- | --- | --- | --- | --- | --- |
| 1 | Al-mousily, 2022 | M | 3 | 10 | Deceased | Immediate | Thymoglobulin, methylprednisolone, mycophenolate mofetil, tacrolimus, corticosteroids, plasmapheresis |
| 2 | Al-mousily, 2022 | F | 13 | 15 | Deceased | Immediate | Thymoglobulin, tacrolimus, mycophenolate mofetil, corticosteroids, Rituximab |
| 3 | Muñoz, 2017 | M | 1.9 | 11 | Living | NR | Cyclosporine, rituximab, plasmapheresis, statin, ezetimibe |
| 4 | Raina, 2019 | NR | NR | NR | NR | NR | Prednisone, valganciclovir, ketoconazole, Bactrim, tacrolimus, amlodipine |
| 5 | Raina, 2019 | NR | NR | NR | NR | NR | None |
| 6 | Raina, 2019 | NR | NR | NR | NR | NR | None |
| 7 | Shah, 2019 | F | 2 | 5 | Deceased | Immediate | Corticosteroids, azathioprine, rituximab, plasmapheresis |
| 8 | Shah, 2019 | F | 2 | 4 | Deceased | Immediate | Corticosteroids, rituximab, plasmapheresis |
| 9 | Shah, 2019 | M | 1.8 | 3.5 | Deceased | Immediate | Corticosteroids, rituximab, cyclosporine, abatacept, plasmapheresis |
| 10 | Shah, 2019 | F | 7 | 9.5 | Living | Immediate | Corticosteroids, rituximab, cyclophosphamide, plasmapheresis |
| 11 | Shah, 2019 | M | 7 | 10 | Deceased | Immediate | Corticosteroids, rituximab, plasmapheresis |
| 12 | Shah, 2019 | F | 4 | 9.5 | Deceased | Immediate | Corticosteroids, rituximab, plasmapheresis |
| 13 | Shah, 2019 | M | 2 | 5 | Living | Immediate | Corticosteroids, plasmapheresis |
| 14 | Kazi, 2023 | NR | NR | 7 | NR | Immediate | Plasmapheresis, rituximab |
| 15 | Kazi, 2023 | NR | NR | 10 | NR | Immediate | Plasmapheresis, Rituximab |
| 16 | Kazi, 2023 | NR | NR | 7 | NR | Delayed (40 months) | Plasmapheresis, Rituximab |
| 17 | Kazi, 2023 | NR | NR | 10 | NR | immediate | Plasmapheresis, Rituximab |
| 18 | McKay, 2022 | F | 2 | 7 | NR | Immediate | Methylprednisolone, Rituximab, plasmapheresis |
| 19 | McKay, 2022 | M | 4 | 15 | NR | Immediate | Rituximab, plasmapheresis |
| 20 | McKay, 2022 | F | 4 | 13 | NR | Immediate | Rituximab, plasmapheresis |
| 21 | McKay, 2022 | F | 10 | 16 | NR | Immediate | Rituximab, plasmapheresis |
| 22 | Morey, 2023 | NR | NR | NR | Deceased | Immediate | Plasmapheresis, immunosuppression, pulse steroids, cyclophosphamide, B cell depletion |
| 23 | Morey, 2023 | NR | NR | NR | Deceased | Immediate | Plasmapheresis, immunosuppression, pulse steroids, cyclophosphamide, B cell depletion |
| 24 | Morey, 2023 | NR | NR | NR | Deceased | Immediate | Plasmapheresis, immunosuppression, pulse steroids, cyclophosphamide, B cell depletion |
| 25 | Fisher, 2021 | F | NR | 12 | Deceased | Immediate | Plasmapheresis, Rituximab, corticosteroids, Ofatumumab |
| Summary of reported characteristics  Category or Subcategory: n/total (%); median (IQR) (if applicable) | Al-mousily, 2022: 2/25 (8)  Muñoz, 2017: 1/25 (4)  Raina, 2019: 3/25 (12)  Shah, 2019: 7/25 (28)  Kazi, 2023: 4/25 (16)  McKay, 2022: 4/25 (16)  Morey, 2023: 3/25 (12)  Fisher, 2021: 1/25 (4) | F: 9/25 (36)  M: 6/25 (24)  NR: 10/25 (40) | Age was reported: 14/25 (56); median age: 3.5 years (IQR 2.0-6.3 years)  NR: 11/25 (44) | Age was reported: 19/25 (76);  median age: 10.0 years (IQR 7.0-11.5 years)  NR: 6/25 (24) | Living: 3/25 (12)  Deceased: 11/25 (44)  NR: 11/25 (44) | Immediate: 20/25 (80)  Delayed: 1/25 (4)  NR: 4/25 (16) | EXTRACORPOREAL / IMMUNOADSORPTIVE TREATMENTS: 21/25 (84)   - Plasmapheresis: 21/25 (84)   IMMUNOSUPPRESSIVE / IMMUNOMODULATORY THERAPIES: 23/25 (92)  *Corticosteroids:* 15/25 (60)   - “steroids,” “pulse steroids,” “corticosteroids,” “methylprednisolone,” or “prednisone”: 15/25 (60)   *B-cell-depleting agents,* 20/25 (80)   - Rituximab: 17/25 (68) - “B cell depletion”: 3/25 (12)   *Calcineurin inhibitors:* 6/25 (24)   - Tacrolimus: 3/25 (12) - Cyclosporine: 2/25 (8) - Ofatumumab: 1/25 (4)   *Antimetabolites,* 3/25 (12)   - Mycophenolate mofetil: 2/25 (8) - Azathioprine: 1/25 (4)   *Cytotoxic agents*: 4/25 (16)   - Cyclophosphamide: 4/25 (16)   *T-cell-depleting agents:* 2/25 (8)   - Thymoglobulin: 2/25 (8)   *Co-stimulatory blockade:* 1/25 (4)   - Abatacept: 1/25 (4)   *Unspecified immunosuppressive treatments:* 3/25 (12)   - “Immunosuppression”: 3/25 (12)   OTHER / ADJUNCTIVE / SUPPORTIVE THERAPIES: 2/25 (8)  *Lipid management:* 1/25 (4)   - Statin: 1/25 (4) - Ezetimibe: 1/25 (4)   *Blood pressure management:* 1/25 (4)   - Amlodipine: 1/25 (4)   *Antimicrobials:* 1/25 (4)   - Bactrim: 1/25 (4) - Ketoconazole: 1/25 (4) - Valganciclovir: 1/25 (4) |

This table lists the following characteristics for each patient included in the meta-analysis: Study first author last name and year (identifying the original study from which the patient’s data was extracted for this meta-analysis), patient sex (recorded as “F” for female or “M” for male), age at time of initial diagnosis of focal segmental glomerulosclerosis (FSGS), age at the time of receiving the kidney transplant that preceded the FSGS recurrence that was treated with low-density lipoprotein apheresis (LDL-A) in the original studies, transplant donor type to indicate whether the transplanted kidney came from a living or deceased donor, classification of the amount of time that passed between the kidney transplant and the diagnosis of recurrent FSGS (rFSGS) as either “immediate” or “delayed” (defined as less than 2 weeks between transplant and recurrence, or more than 2 weeks, respectively), and a list of any treatments the patient received for FSGS recurrence prior to beginning LDL-A therapy. “NR” = not reported by original study. The proportion and percentage of patients in this meta-analysis were calculated for each category/subcategory of characteristic reported. Median and interquartile range (IQR) were calculated to represent average age at initial diagnosis and at transplant.
