# Supplementary material for: Low-density lipoprotein apheresis for recurrent focal segmental glomerulosclerosis in pediatric kidney transplant recipients: a systematic review and meta-analysis
Source: Pediatr Nephrol. 2026 Feb 11;41(9):2849–61. doi: 10.1007/s00467-025-07143-z (PMC13424331; doi:10.1007/s00467-025-07143-z)
Supplement: Supplementary file 3 — (DOCX 18.4 KB) [file 467_2025_7143_MOESM3_ESM.docx]

**Supplemental Table S3. LDL-apheresis protocol details for time to treatment, total number of LDL-apheresis sessions, duration of LDL-A treatment, and concomitant treatments, by patient.**

| **Patient** | **Time from transplant to LDL-A initiation (months)** | **Total number of LDL-A sessions** | **LDL-A duration (weeks)** | **Concomitant treatment(s)** |
| --- | --- | --- | --- | --- |
| 1 | 2 | 12 | 9 |  |
| 2 | 0.25 | 12 | 9 |  |
| 3 | 36 | 12 | 9 | Methylprednisolone (1 mg/kg/day) from the sixth session; cyclosporine and mycophenolate were maintained. |
| 4 | NR | 12 | 9 | Prednisone (before 2^nd^ session), cyclophosphamine (before 3^rd^ session), tacrolimus (before 4^th^ session), amlodipine (before 7^th^ session), mycophenolate mofetil (before 9^th^ session), metolazone (before 11^th^ session) |
| 5 | NR | 12 | 9 |  |
| 6 | NR | 12 | 9 | Mycophenolate mofetil (before 10^th^ session) |
| 7 | 8 | 17-20 | 14 | IV methylprednisolone (10-20 mg/kg for a single maximum dose of 1 g) once per week for the final six weeks |
| 8 | 4 | 12-15 | 9 | IV methylprednisolone (10-20 mg/kg for a single maximum dose of 1 g) once per week for the final six weeks |
| 9 | 3 | 25-28 | 22 | IV methylprednisolone (10-20 mg/kg for a single maximum dose of 1 g) once per week for the final six weeks |
| 10 | 5 | 19-22 | 16 | IV methylprednisolone (10-20 mg/kg for a single maximum dose of 1 g) once per week for the final six weeks |
| 11 | 0.25 | 12-15 | 9 | IV methylprednisolone (10-20 mg/kg for a single maximum dose of 1 g) once per week for the final six weeks |
| 12 | 18 | 12-15 | 9 | IV methylprednisolone (10-20 mg/kg for a single maximum dose of 1 g) once per week for the final six weeks |
| 13 | 2 | 12-15 | 9 | IV methylprednisolone (10-20 mg/kg for a single maximum dose of 1 g) once per week for the final six weeks |
| 14 | 0 | 12 | 9 |  |
| 15 | 0 | 24 | 18 |  |
| 16 | 40 | 12 | 9 |  |
| 17 | 0 | 12 | 9 |  |
| 18 | 17 | 12-15 | 9 | IV methylprednisolone (10-20 mg/kg for a single maximum dose of 1 g) once per week for the final six weeks |
| 19 | 20 | 12-15 | 9 | IV methylprednisolone (10-20 mg/kg for a single maximum dose of 1 g) once per week for the final six weeks |
| 20 | 0.1 | 12-15 | 9 | IV methylprednisolone (10-20 mg/kg for a single maximum dose of 1 g) once per week for the final six weeks |
| 21 | 3 | 12-15 | 9 | IV methylprednisolone (10-20 mg/kg for a single maximum dose of 1 g) once per week for the final six weeks |
| 22 | NR | 84 | 124 | “Baseline immunosuppression” |
| 23 | NR | 29 | 44 | “Baseline immunosuppression” |
| 24 | NR | 41 | 52 | “Baseline immunosuppression” |
| 25 | NR | 10 | 20 | “high-dose intravenous steroids” |

LDL-apheresis (LDL-A) protocols are detailed in the table by patient, including the time to treatment (“Time from transplant to LDL-A initiation”), the total number of LDL-A sessions administered, and the duration of weeks over which the LDL-A sessions were administered (“LDL-A Duration”). Sessions and duration were recorded as either a number or a range for each patient depending on how the values were reported in the original study. Missing values are indicated by “NR” (NR = not reported). Treatments received in combination with LDL-A as part of the protocol, or any treatment received during the duration of LDL-A (“Concomitant treatment(s)”), are listed by patient as specific protocol definitions, specific treatment details/notes for individual patients, or as non-specific protocol descriptions (indicated by phrases in quotation marks), all recorded as written in the original study.
