# Supplementary material for: Low-density lipoprotein apheresis for recurrent focal segmental glomerulosclerosis in pediatric kidney transplant recipients: a systematic review and meta-analysis
Source: Pediatr Nephrol. 2026 Feb 11;41(9):2849–61. doi: 10.1007/s00467-025-07143-z (PMC13424331; doi:10.1007/s00467-025-07143-z)
Supplement: Supplementary file 4 — (DOCX 23.0 KB) [file 467_2025_7143_MOESM4_ESM.docx]

**Supplemental Table S4. Individual patient data for remission status, time to latest follow-up measurement of lab values, and baseline and final lab values for serum creatinine, serum albumin, urine protein-creatinine ratio (UPCR), and estimated glomerular filtration rate (eGFR).**

| **Patient** | **Time from LDL-A initiation to measurement of final lab values (months)** | **Serum creatinine at LDL-A initiation (mg/dL)** | **Serum creatinine at latest follow-up (mg/dL)** | **Serum albumin at LDL-A initiation (g/dL)** | **Serum albumin at latest follow-up (g/dL)** | **Urine protein-creatinine ratio (UPCR) at LDL-A initiation (g/g)** | **Urine protein-creatinine ratio (UPCR) at latest follow-up (g/g)** | **eGFR at LDL-A initiation (ml/min/1.73 m^2^)** | **eGFR at latest follow-up (ml/min/1.73 m^2^)** | **Remission status at latest follow-up for UPCR** |
| --- | --- | --- | --- | --- | --- | --- | --- | --- | --- | --- |
| 1 | 36 | NR | 0.9 | NR | NR | 7.7 | 0 | NR | NR | Complete remission |
| 2 | 6 | NR | 0.9 | NR | NR | 2.5 | 0.8 | NR | NR | Partial remission |
| 3 | 24 | NR | 1 | NR | NR | “Active nephrotic syndrome” | 0.2-0.5 | NR | “Correct graft function” | Partial remission |
| 4 | 2.25 | 0.8 | 1.3 | NR | NR | 19.52 | 25.58 | 76.6 | 47.1 | No remission |
| 5 | 1 | 2.1 | 0.4 | NR | NR | 4.78 | 3.66 | 84.7 | 129.8 | No remission |
| 6 | 24 | 0.8 | 1.1 | NR | NR | 1.09 | 0.34 | 78.0 | 71.7 | Partial remission |
| 7 | UPCR: 6  eGFR: 25  Serum albumin: 3.7 | NR | NR | 2.1 | 4.3 | 18.2 | 0.2 | 50.3 | 98.0 | Complete remission |
| 8 | UPCR: 8  eGFR: 16  Serum albumin: 6.9 | NR | NR | 2.4 | 3.4 | 20.9 | 0.3 | 115.1 | 169.3 | Partial remission |
| 9 | UPCR: 6  eGFR: 20  Serum albumin: 6 | NR | NR | 2.6 | 3.5 | 18.1 | 0.43 | 182.1 | 81.8 | Partial remission |
| 10 | UPC ratio: 7.5  eGFR: 12  Serum albumin: 7.5 | NR | NR | 2.5 | 4 | 8.6 | 1.8 | 47.0 | 60.0 | Partial remission |
| 11 | 6 | NR | NR | 3 | 3.5 | 6.9 | 0.19 | 20.8 | 105.7 | Complete remission |
| 12 | UPCR: 6  eGFR: 10  Serum albumin: 8.1 | NR | NR | 3.3 | 3.8 | 4.7 | 0.42 | 40.0 | 107.4 | Partial remission |
| 13 | UPCR: 4.2  eGFR: 4  Serum albumin: 6.7 | NR | NR | 2.5 | 3.5 | 59.8 | 0.15 | 46.0 | 105.9 | Complete remission |
| 14 | 30 | 0.6 | 0.6 | 1.6 | 4.6 | 17.5 | <0.2 | 64.2 | 81.2 | Complete remission |
| 15 | 24 | 2.7 | 1.1 | 2.5 | 4.0 | 34.8 | 0.7 | 27.7 | 54.1 | Partial remission |
| 16 | 2 | 1.1 | 0.5 | 2.6 | 3.2 | 15.2 | 1.6 | 47.7 | 89.3 | Partial remission |
| 17 | 1 | 0.3 | 0.5 | 2.2 | 4.2 | 50.1 | <0.2 | 127 | 91.9 | Complete remission |
| 18 | 19 | NR | NR | NR | NR | NR | <0.2 | NR | NR | Complete remission |
| 19 | 16 | NR | NR | NR | NR | NR | <0.2 | NR | NR | Complete remission |
| 20 | 11 | NR | NR | NR | NR | NR | >2.0 | NR | “Stable graft function” | No remission |
| 21 | 8 | NR | NR | NR | NR | NR | 0.2-2 | NR | “Stable graft function” | Partial remission |
| 22 | 31 | NR | NR | 2.8 | 3.0 | 10 | 4.6 | 25 | 30 | No remission |
| 23 | 50 | NR | NR | 2.8 | 4.7 | 11 | <0.2 | 14 | 80 | Complete remission |
| 24 | 38 | NR | NR | 3.6 | 4.7 | 7.7 | <0.2 | 12 | 55 | Complete remission |
| 25 | 15 | NR | NR | 2.4 | NR | >0.2 | NR | 88 | 0, “Graft loss” | No remission |

Lab values for serum creatinine, serum albumin, estimated glomerular filtration rate (eGFR), and urine protein-creatinine ratio (UPCR) at LDL-apheresis initiation (representing baseline/initial values) and at the time of latest follow-up at which a specific lab value was measured and reported (representing final values). Time to latest follow-up measurement/final reported lab value from the time of initiating LDL-A treatment is listed by patient as either a single value that indicates all lab values were measured at the same follow-up point, or were specified for each lab test if the latest reported lab values differed between tests/were measured on different days. Numerical lab values, ranges, or written interpretations of lab values (indicated by phrases within quotation marks) were extracted from included studies for each patient, depending on how outcomes were reported and defined in the original studies. Missing lab values are indicated in the table as “NR” (NR = not reported). Remission status was determined by the latest reported UPCR value or range for each patient, based on the following definitions: UPCR $\leq$ 0.2 g/g = “Complete remission,” UPCR between 0.2 and 2.0 g/g = “Partial remission,” and UPCR $\geq$ 2.0 g/g = “No remission.”
