# Supplementary material for: Low-density lipoprotein apheresis for recurrent focal segmental glomerulosclerosis in pediatric kidney transplant recipients: a systematic review and meta-analysis
Source: Pediatr Nephrol. 2026 Feb 11;41(9):2849–61. doi: 10.1007/s00467-025-07143-z (PMC13424331; doi:10.1007/s00467-025-07143-z)
Supplement: Supplementary file 5 — (DOCX 15.0 KB) [file 467_2025_7143_MOESM5_ESM.docx]

| **#** | **Search** |
| --- | --- |
| 1 | exp Child/ |
| 2 | exp Adolescent/ |
| 3 | exp Pediatrics/ |
| 4 | (child or children* or childhood or kid or kids or adolescent or adolescents or adolescence or preteen* or teenager* or teen or teens or toddler or toddlers or preschool* or school-age* or girl or girls or girlhood or boy or boys or boyhood or youth or pediatric or pediatrics).ti,ab. |
| 5 | or/1-4 |
| 6 | exp Glomerulosclerosis, Focal Segmental/ |
| 7 | exp Kidney Transplantation/ |
| 8 | Nephrotic Syndrome/ |
| 9 | ((Kidney* or renal) adj2 (transplant* or graft* or posttransplant*)).ti,ab. |
| 10 | (focal-segmental-glomerulosclerosis or Focal-segmental-glomerular-sclerosis or fsgs or steroid-resistant-nephrotic-syndrome).ti,ab. |
| 11 | or/6-10 |
| 12 | exp Lipoproteins, LDL/ |
| 13 | (LDL-apheresis or LDL?A or lipoprotein-apheresis or lipoprotein?a or lp?a or therapeutic-apheresis or apheresis-therap* or apheresis-treatment*).ti,ab. |
| 14 | 12 or 13 |
| 15 | 5 and 11 and 14 |
| 16 | 11 and 14 |
