# Supplementary material for: Low-density lipoprotein apheresis for recurrent focal segmental glomerulosclerosis in pediatric kidney transplant recipients: a systematic review and meta-analysis
Source: Pediatr Nephrol. 2026 Feb 11;41(9):2849–61. doi: 10.1007/s00467-025-07143-z (PMC13424331; doi:10.1007/s00467-025-07143-z)
Supplement: Supplementary file 6 — (PPTX 386 KB) [file 467_2025_7143_MOESM6_ESM.pptx]

## Slide 1
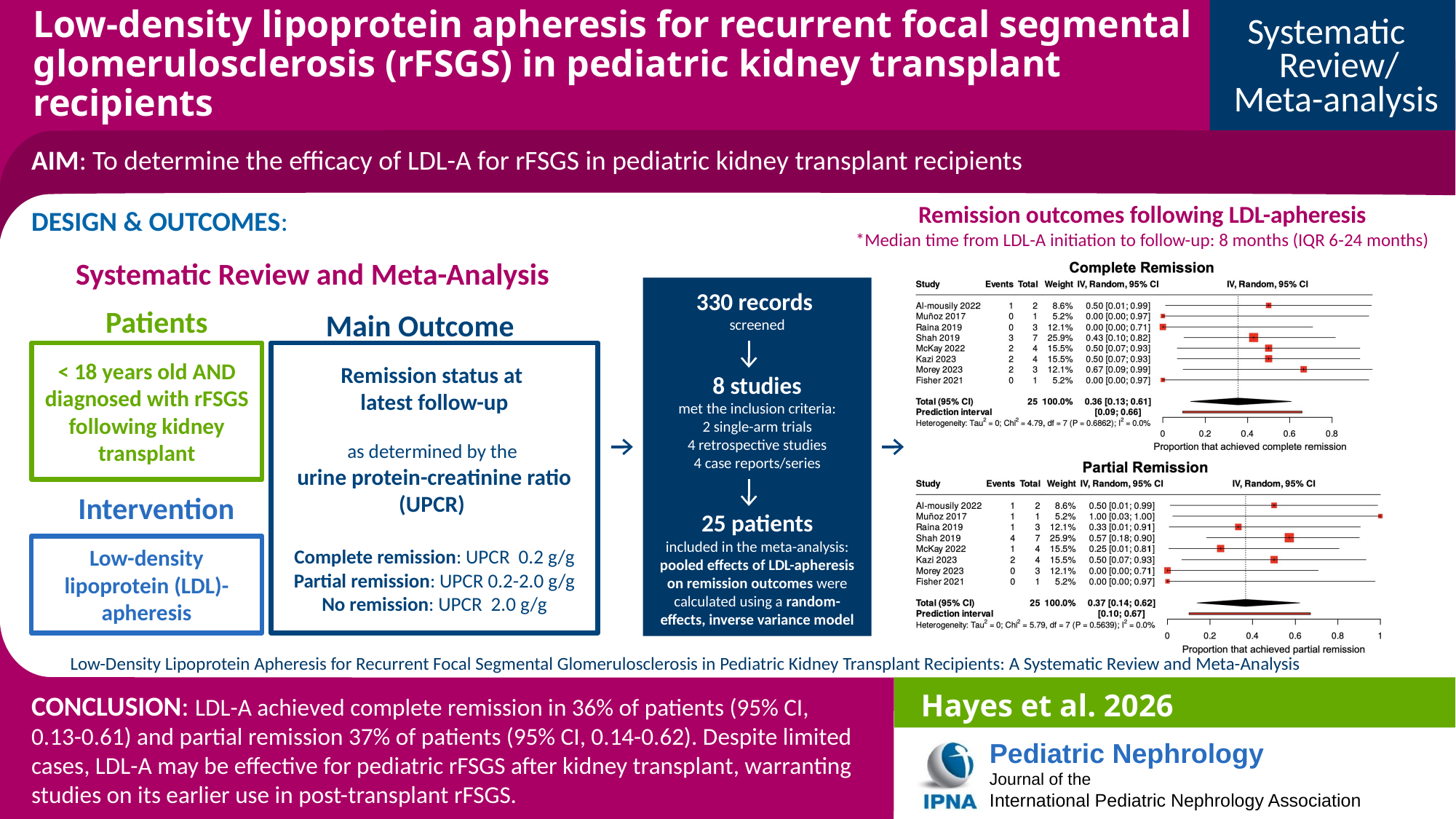

Low-density lipoprotein apheresis for recurrent focal segmental glomerulosclerosis (rFSGS) in pediatric kidney transplant recipients
AIM: To determine the efficacy of LDL-A for rFSGS in pediatric kidney transplant recipients
Remission outcomes following LDL-apheresis
*Median time from LDL-A initiation to follow-up: 8 months (IQR 6-24 months)
DESIGN & OUTCOMES:
Systematic Review and Meta-Analysis
330 records
screened
8 studies
met the inclusion criteria:
2 single-arm trials
4 retrospective studies
4 case reports/series
25 patients
included in the meta-analysis: pooled effects of LDL-apheresis on remission outcomes were calculated using a random-effects, inverse variance model
Patients
Main Outcome
< 18 years old AND diagnosed with rFSGS following kidney transplant
Intervention
Low-density lipoprotein (LDL)-apheresis
Low-Density Lipoprotein Apheresis for Recurrent Focal Segmental Glomerulosclerosis in Pediatric Kidney Transplant Recipients: A Systematic Review and Meta-Analysis
Hayes et al. 2026
CONCLUSION: LDL-A achieved complete remission in 36% of patients (95% CI, 0.13-0.61) and partial remission 37% of patients (95% CI, 0.14-0.62). Despite limited cases, LDL-A may be effective for pediatric rFSGS after kidney transplant, warranting studies on its earlier use in post-transplant rFSGS.
